# Mid-Infrared Photoacoustic Detection of Glucose in Human Skin: Towards Non-Invasive Diagnostics

**DOI:** 10.3390/s16101663

**Published:** 2016-10-10

**Authors:** Jonas Kottmann, Julien M. Rey, Markus W. Sigrist

**Affiliations:** 1 Laser Spectroscopy and Sensing Laboratory, Institute for Quantum Electronics, ETH Zürich, Zürich CH-8093, Switzerland; kottmannjonas@gmail.com (J.K.); reyj@zhaw.ch (J.M.R.); 2 BIOTRONIK AG, Bülach CH-8180, Switzerland; 3 Institute of Applied Mathematics and Physics, Zurich University of Applied Sciences (ZHAW), Winterthur CH-8401, Switzerland

**Keywords:** glucose, quantum cascade laser, spectroscopy, photoacoustic

## Abstract

Diabetes mellitus is a widespread metabolic disease without cure. Great efforts are being made to develop a non-invasive monitoring of the blood glucose level. Various attempts have been made, including a number of non-optical approaches as well as optical techniques involving visible, near- and mid-infrared light. However, no true breakthrough has been achieved so far, i.e., there is no fully non-invasive monitoring device available. Here we present a new study based on mid-infrared spectroscopy and photoacoustic detection. We employ two setups, one with a fiber-coupled photoacoustic (PA) cell and a tunable quantum cascade laser (QCL), and a second setup with two QCLs at different wavelengths combined with PA detection. In both cases, the PA cells are in direct skin contact. The performance is tested with an oral glucose tolerance test. While the first setup often gives reasonable qualitative agreement with ordinary invasive blood glucose measurements, the dual-wavelength approach yields a considerably improved stability and an uncertainty of only ±30 mg/dL of the blood glucose concentration level at a confidence level of 90%. This result is achieved without advanced data treatment such as principal component analysis involving extended wavelength ranges.

## 1. Introduction

Diabetes mellitus is a metabolic disease with a dramatically increasing number of people affected, currently over 400 million people worldwide [1]. There are two types of diabetes, type I (also named insulin dependence or juvenile diabetes) and type II (also non insulin depending or adult onset diabetes). Ninety percent of the patients suffer from diabetes type II. For either case there exists no treatment that could possibly cure this illness. The treatment today consists in surveilling the blood glucose level of the patient to avoid the risk of hypo- and hyperglycemia, assisting in diet adjustment as well as medical therapy. The monitoring of the blood glucose (BG) level requires the taking of a small drop of blood (≤1 μL) with a lancing device—usually from the finger tip 2–4 times daily—put the droplet on a test stripe and directly read the BG value on a glucometer. This procedure is uncomfortable and bears the risk of infections. The BG level of a healthy person under non-fasting conditions fluctuates during the day between approximately 60 and 130 mg/dL (3.3 to 7.15 mmol/L) with maximum levels of up to 250 mg/dL (13.75 mmol/L). The mean level is about 100 mg/dL (5.5 mmol/L) [2].

A non-invasive BG monitoring instrument would ease the life of diabetes patients drastically. However, up to now, no reliable method has been found, and despite intense research and development there is no non-invasive commercial technique or device. Numerous attempts have been explored for in vivo glucose measurements. An overview has been given previously [3], differentiating between invasive, non-invasive and minimal invasive techniques. The non-invasive techniques comprise different types of optical (mainly spectroscopic) methods, and non-optical schemes like electromagnetic, ultrasound, or bioimpedance measurements. So far, most spectroscopic measurements focused on the near-infrared (NIR) region because NIR light can penetrate up to several milimeters into human tissue and reaches blood vessels. Unfortunately, however, the glucose absorption is weak and interferes strongly with other blood and tissue components [4,5,6]. Furthermore, accuracy issues need to be solved before future clinical applications can be envisaged, as reviewed recently by Liu et al. [7].

In contrast, the mid-IR region benefits from strong glucose absorption but at the price of strong water absorption. In recent years, several groups have reported on mid-IR studies of glucose sensing employing quantum cascade lasers. In a first step, Lilienfeld-Toal et al. presented the evidence that it is possible to reach the glucose-containing layer of the skin and that the overall glucose concentration in the investigated skin layer is almost that of blood. They employed two QCLs, one at 1080 cm^−1^ (at the maximum of a glucose absorption band) and a second one at 1066 cm^−1^ between two absorption bands. During an oral glucose tolerance test, absorption signals were recorded from the skin of the forearm of a healthy volunteer with a photoacoustic setup [8]. Later, Pleitez et al. introduced an ultrasound (50–60 kHz) windowless photoacoustic cell and a tunable QCL between 1000 cm^−1^ and 1245 cm^−1^ [9]. The use of a windowless cell minimized the influence of air pressure and temperature variations as well as the buildup of air humidity caused by the direct contact of a cell to the skin. The fast tuning reduced artefacts from arm movements in in vivo measurements. Hence, the signal-to-noise ratio could be improved. The viability of the technique was illustrated with a preliminary test on healthy volunteers and volunteers suffering from diabetes mellitus [10]. Most recently, Pleitez et al. employed another approach based on photothermal deflectometry enhanced by total internal reflection, named TIR-PTD spectroscopy. This technique enables to reach deeper skin layers compared to conventional IR techniques. The use of sophisticated mathematical analyses based on partial least square regression (PLSR) and principal component analysis (PCA) combined with root mean square error of cross validation (RMSECV) enabled to establish a correlation between epidermal- and blood-glucose levels on a type 1 diabetic patient [11].

Guo et al. introduced a new technique of wavelength modulated differential laser photothermal radiometry (WM-DPTR) for noninvasive glucose detection in human skin [12]. They employed two QCLs at two discrete wavelengths, 9.5 μm and 10.4 μm, i.e., near the peak and the baseline of glucose absorption, respectively, modulated out-of-phase. As a first step they presented results from aqueous glucose phantoms in the concentration range 0–440 mg/dL. Later, they demonstrated the feasibility of WM-DPTR by applying the technique to in vitro human blood serum (with glucose concentrations in the physiological range) diffused into one milimeter-thick human skin samples [13].

Liakat et al. [14] presented a study using a hollow-fiber coupled pulsed tunable QCL (8–10 μm) directed to the human palm. Backscattered radiation from the skin was collected with a bundle of six fibers and analyzed in the glucose-specific wavelength region around 1075–1085 cm^−1^. Second derivative spectra, chemo-metric methods employing partial least squares regression (PLSR) and Clarke grids were used to analyze the data from three human subjects. On average, clinically accurate predictions of the glucose levels were obtained in 84% of the time.

Numerous other approaches have been reported, yet most studies were performed in vitro. Most recently, Kino et al. [15] used a Fourier transform infrared (FTIR) spectrometer and attenuated total reflection (ATR) with a hollow-cored fiber and a trapezoidal multi-reflection ATR prism to record in vivo spectra around 1155 cm^−1^ of human inner lip mucosa. They achieved measurement errors for blood glucose levels of less than 20%.

**Figure 1:**
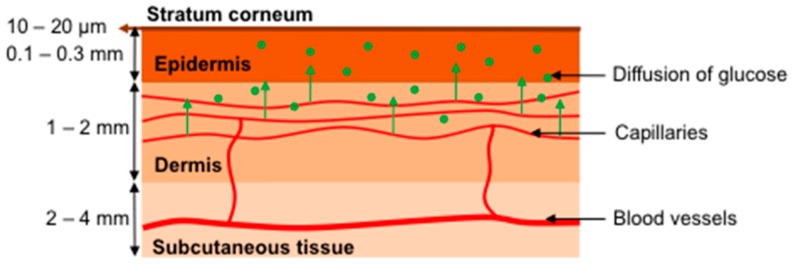
Schematic of the human skin with stratum corneum, epidermis, dermis and subcutaneous tissue.

As mentioned above, glucose sensing through human skin is impeded by strong water absorption which reduces the penetration depth of mid-IR light to below 100 μm. Hence, the glucose content has to be monitored in the interstitial fluid (ISF) rather than in the blood itself, since neither blood vessels nor capillaries can be reached. The situation is illustrated in Figure 1, which shows a cross section through the human skin layers with stratum corneum on top (thickness 10–20 μm), epidermis (0.1–0.3 mm), dermis (1–2 mm) and subcutaneous tissue (2–4 mm).

The subcutaneous tissue contains the larger blood vessels and nerves while the epidermis houses a dense net of blood capillaries and supplies the non-vascular epidermis with nutrition via diffusion. The outer stratum corneum (horny layer) is rather dry and contains only about 10–20% water. The nutrition of the living cells (essentially keratinocytes) in the epidermis (stratum spinosum [10]) occurs exclusively through glucose diffusion via the ISF. Hence, for in vivo measurements in the ISF within the epidermis, time delays of 5–15 min between blood glucose and ISF measurements—as indicated by previous studies—are to be expected [16,17,18,19,20,21].

In contrast to previous studies, we focus on individual time-resolved in vivo measurements in this paper as would be the case in a practical application, and we abstain from the application of advanced chemometric methods across the wavelength range of interest to evaluate the data. An oral glucose tolerance test (OGTT)—which is usually employed for the diagnosis of diabetes mellitus—is taken as the test procedure to validate the PA measurements. As discussed in the following, we use a tunable quantum cascade laser (QCL), or, in a second approach, two QCLs at selected fixed wavelengths and photoacoustic (PA) detection for our measurements.

## 2. Photoacoustic Detection: Theory

The PA effect for solid samples is well described by the Rosencwaig-Gersho model (so-called gas piston model [22]). For the case of indirect detection of the acoustic wave in a gas volume above the sample (as in our case [23]), the model distinguishes six different cases depending on the thickness *L* of the sample, the optical penetration depth (i.e., absorption depth) *µ*_a_, and the thermal diffusion length *µ*_s_. The parameters *µ*_a_ and *µ*_s_ are given by Equation (1):

(1) $$\mu_{a}\left(\lambda \right)=\frac{1}{\alpha \left(\lambda \right)}\text{ and }\mu_{s}={\left(\frac{D}{\pi \cdot f}\right)}^{1/2}$$

where *α*(*λ*) denotes the absorption coefficient of the sample at the wavelength *λ*, *D* is the thermal diffusivity of the sample and *f* represents the modulation frequency of the laser. The thermal diffusivity *D* is given by Equation (2):

(2) $$D=\frac{k}{\rho \cdot C}$$

where *k* is the thermal conductivity, *ρ* the density and *C* the specific heat.

**Figure 2:**
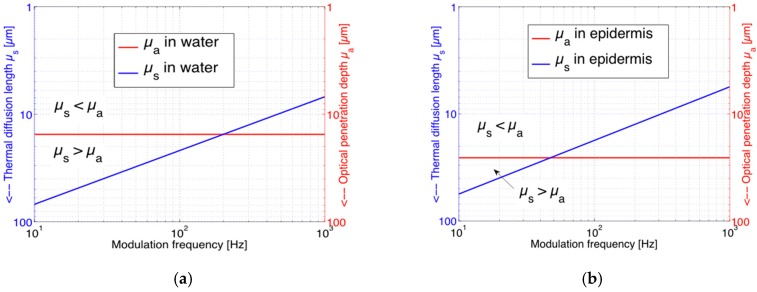
Dependence of the thermal diffusion length *µ*_s_ (blue) and the optical penetration depth *µ*_a_ (red) on modulation frequency *f* for the wavelength range between 1000 and 1100 cm^−1^: (**a**) For water and (**b**) for the epidermis. Obviously, *µ*_a_ does not depend on *f*. The comparison between *µ*_a_ and *µ*_s_ allows a classification of different photoacoustic (PA) regimes.

The only interesting case for our application is the optically and thermally thick case, i.e., for the condition *L* >> *µ*_a_ > *µ*_s_. In this case the amplitude *S* of the PA signal is given by [24], presented here as Equation (3):

(3) $$S_{PA}\propto \frac{\alpha \left(\lambda \right)\cdot I_{0}\left(\lambda \right)\cdot p_{0}}{V\cdot f^{3/2}}$$

where *I*_0_(*λ*) represents the incident laser intensity and *V* the enclosed gas volume of the PA cell with gas pressure *p*_0_. Only in this case, the PA signal is proportional to the absorption coefficient *α*(*λ*) and hence to the glucose content of the skin. The requirement of a large sample thickness *L* is automatically fulfilled as the absorption depth in skin is small. The additional requirement *µ*_a_ > *µ*_s_ can be fulfilled by choosing an appropriate modulation frequency *f*. This is illustrated in Figure 2 which shows the calculated data of *µ*_a_ and *µ*_s_ in dependence of the modulation frequency *f* for water (Figure 2a) and for the epidermis (Figure 2b).

As expected, *µ*_a_ is independent of *f* whereas *μ*_s_ shows the *f* ^−1/2^-dependence. The data used for the calculation are given in Table 1 with material constants taken from [25,26,27].

For the dry surface layer of the human skin, the stratum corneum, we used an absorption coefficient of 0.1·*α* of that of water because stratum corneum only contains about 10% of water whereas the epidermis contains approximately 60%, hence its absorption coefficient is taken as 0.6·*α* of the pure water absorption. Thereby, absorption by other tissue components are neglected. As Figure 2a indicates, a modulation frequency *f* above 200 Hz would have to be chosen to fulfill the requirement of *µ*_a_ > *µ*_s_ in water, whereas approx. 50 Hz appears sufficient for the epidermis. Usually we used *f* = 130 Hz for our experiments on skin to warrant *µ*_a_ > *µ*_s_ without unnecessarily reducing the PA signal amplitude which scales with *f*^−3/2^ (see Equation (3)). In fact, for a skin configuration of a 20 µm thick stratum corneum layer on top of the epidermis, the optical penetration depth 1/*α* might exceed 45 µm and one could even consider choosing *f* down to 20 Hz and could still expect a linear dependence of the PA signal on *α*.

## 3. Experimental Arrangements

### 3.1. Setup with Fiber-Coupled EC-QCL

The first experimental setup essentially involves an external cavity quantum cascade laser (EC-QCL, Daylight Solutions, San Diego, CA, USA) coupled to a photoacoustic cell and has been described previously [28]. The continuous wave (cw) QCL is tunable in the wavelength range between 1005 cm^−1^ and 1100 cm^−1^. This range covers two distinct peak absorptions of glucose at 1034 cm^−1^ and 1080 cm^−1^. The laser beam is chopped with a mechanical chopper and transferred to a home-made photoacoustic (PA) cell. The cell is put into direct contact with the skin surface at the inner human forearm. The compact PA cell has been described in detail previously [28]. It contains a conically-shaped gas volume of 35 mm^3^ for detecting the generated PA signal in the gas phase above the sample surface. A 0.5 mm-high ring structure, called “pressure seal” on the sample side, i.e., the skin surface for in vivo measurements, warrants a good and stable contact. A conventional miniature electret microphone (FG-23329-P07, Knowles Electronics, LLC, Itasca, IL, USA) with a responsivity of 22 mW/Pa is used. The detected signal gets preamplified and is measured with a lock-in amplifier (SR830, Stanford Research Systems, Inc., Sunnyvale, CA, USA) before being recorded with a computer. In order to control and compensate variations in relative humidity (RH) and temperature T, a RH-T sensor (SHT21, Sensirion, Staefa, Switzerland) is also incorporated into the cell. Furthermore, the cell is ventilated by nitrogen to prevent any water condensation during the measurements. The cell thus contains a stable mixture of primarily nitrogen with traces of ambient air and water vapor due to transpiration of the skin. The system enables continuous long-time measurements. The fiber-coupled PA cell is fixed to the inner forearm of a volunteer with a hook-and-loop tape. For the in vivo studies presented here the cell is connected to an infrared silver halide fiber (CeramOptec GmbH, Bonn, Germany), which transfers the laser beam to the sample. Unlike a previous arrangement with a slightly different PA cell design [29] and with free laser beam access, the fiber-coupled cell does not require any further fixation during measurements. The QCL power is reduced to <6 mW in order to obey the maximum permissible exposure limit of 1 mW/mm^2^ (valid for the irradiation of a 10 mm^2^ skin surface of up to 8 h [30]) for an irradiated skin area of 3 mm^2^ at 50% duty cycle.

For the in vivo studies involving volunteers, the approval of the ethics committee of the ETH Zürich (EK 2010-N-46) was obtained.

### 3.2. Setup with Two QCLs at Preselected Fixed Wavelengths

**Figure 3:**
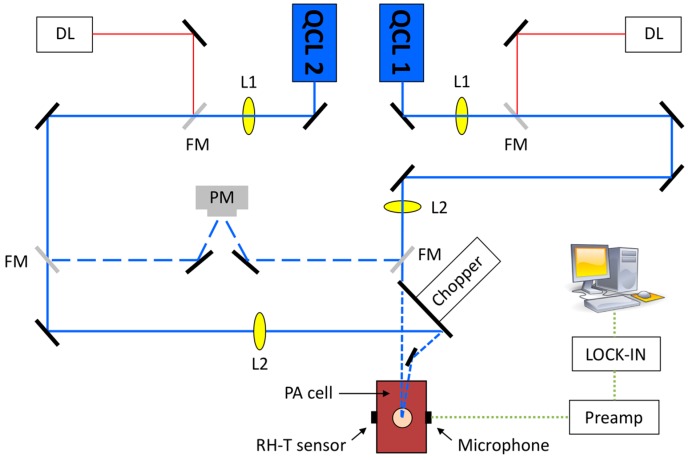
Experimental arrangement with two fixed-wavelength quantum cascade lasers (QCLs) and the N_2_-ventilated PA cell with free laser beam access. A power meter (PM) is employed for power normalization. L1, L2: lenses; RH-T sensor: Sensors for relative humidity (RH) and temperature (T), respectively; DL: diode lasers for alignment purposes.

The second arrangement is presented in Figure 3. It involves the cw EC-QCL (Daylight Solutions, labeled QCL1) employed in the study presented above and a second pulsed EC-QCL (Daylight Solutions, called QCL2) with a pulse repetition rate of 100 kHz, a pulsewidth of 500 ns, a tuning range between 1130 and 1310 cm^−1^, a spectral resolution of 0.9 cm^−1^ when tuned via the external cavity and an average power of <32 mW, decreasing to approximately 8 mW at the preselected wavelength of 1180 cm^−1^. Hence, one EC-QCL covers a wavelength range of strong glucose absorption with the absorption peaks at 1034 cm^−1^ and 1080 cm^−1^, while the second EC-QCL range contains the glucose absorption peak at 1152 cm^−1^ but also a region of negligible glucose absorption around 1180 cm^−1^ and a region of weak absorption above 1190 cm^−1^. However, the studies reported here have been performed with the two QCLs at preselected fixed wavelengths. The beams of the two QCLs are alternately directed to the power meter (PM) or the PA cell by flipping mirrors (FM). Just in front of the PA cell both lasers are modulated by a mechanical chopper.

The QCL1 beam passes through the chopper blade slits and reaches the PA cell, while the QCL2 beam is blocked by the flipping mirror. Alternatively, when the QCL1 beam is blocked by the other flipping mirror, the QCL2 beam is deflected from the gold-coated chopper wheel and reaches the PA cell. Hence, at any time either the QCL1 beam or the QCL2 beam is directed to the PA cell, respectively. This out-of-phase wavelength modulation measurement method allows in particular compensating for long-term drifts induced by either sample variations or measurement conditions. Short-time fluctuations caused by surrounding noise or quick movements are significantly reduced but cannot be fully compensated. The lenses L1 and L2 are used for shaping the QCL beams. This arrangement does not involve the fiber-coupled PA cell but the N_2_-ventilated PA cell described previously with free laser beam access [29]. The microphone signals are amplified by a preamplifier and fed to a lock-in amplifier for data acquisition. The free laser beam access results in laser spot areas on the skin surface that are smaller by approximately a factor of four compared to the fiber-coupled cell. Hence, the available laser power of 8 mW has to be reduced to 2 mW, resulting in 1 mW after modulation, to obey the maximum permissible exposure limit for skin. This applies to both QCL lasers.

The ultimate goal of the above presented setup would be to direct both laser beams simultaneously out-of-phase into the PA cell. For this scheme, the laser power of the two lasers can be adjusted to generate a similar PA signal under baseline conditions of the sample. Hence, a close-to-zero baseline signal is generated when out-of-phase laser beams are directed into the PA cell. Thus, when measuring the time dependence of the PA signal any absorption change of the sample at one of the chosen wavelengths induces an increase of the PA signal. This method allows compensating for short and long-term fluctuations of the PA signals. However, due to the permanent irradiation of the human skin, the laser power has to be further reduced to not exceed the maximum permitted limit, and consequently higher detection limits for glucose are obtained. Thus, for the results presented here, the QCL1 and QCL2 beams are directed into the PA cell sequentially, e.g., QCL1 beam for 30 s and then QCL2 beam for 30 s, and so on. This configuration cancels long-term but not all short term fluctuations, yet permits doubled laser powers to be applied since only one beam at the time reaches the skin.

## 4. Results and Discussion

### 4.1. Fiber-Coupled EC-QCL

First measurements with a PA cell with a thin diamond membrane cover were performed with aqueous solutions of glucose and gels [23], with keratinocyte solutions [29], and with human epidermal skin samples [3]. Later the PA cell was modified to the current configuration to enable in vivo measurements at different body sites [28]. In vivo spectra of the skin at the human forearm recorded with an FTIR-ATR spectrometer (Fourier Transform Infrared Attenuated Total Reflection) as well as spectra of epidermal tissue recorded with the tunable EC-QCL [3] indicated that water and the epidermal constituents nucleic acids, carbohydrated lipids, proteins and albumin show characteristic absorptions by C–OP, CC and PO_2_^−^ vibrations [28,31,32,33,34] in the wavelength range between 1000 and 1100 cm^−1^. However, additional spectra recorded of epidermis with and without glucose content indicate that the epidermal constituents do not impair glucose detection [3]. Whereas a detection limit (±1σ standard deviation) of 30 mg/dL was found for aqueous glucose solutions [23], this limit increased to 100 mg/dL for skin samples mainly due to the mechanical instability of the <100 µm thin epidermis layers [3]. The diffusion of glucose from an underlying solution into the skin samples could directly be observed and took only 1–2 min [3]. These time-resolved PA measurements of the glucose content in skin samples confirmed that in vivo glucose measurements through the stratum corneum are feasible.

**Figure 4:**
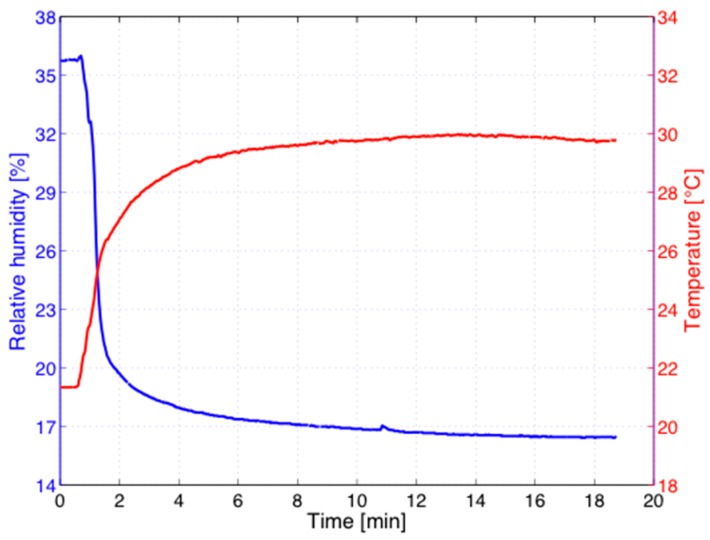
Temporal evolution of the relative humidity RH (blue) and the temperature T (red) during a continuous in vivo measurement at the human forearm with the N_2_-ventilated open-ended PA cell.

In vivo measurements suffer from influences normally not encountered in a laboratory environment. During in vivo studies not only the transdermal water loss through passive diffusion but also transpiration, especially above 30 °C, provoke the evaporation of water into the coupling gas of the PA chamber. Without ventilation of the cell, the uncontrolled increase of the RH may cause problems such as a reduction of the microphone responsivity or even condensation. An example is shown in Figure 4, where the temperature T and the relative humidity (RH) recorded in the gas volume of the PA cell attached directly to the human forearm are plotted. As demonstrated in Figure 4, however, the application of a continuous N_2_ flow with a flow rate of 10 standard cubic centimeter per minute (sccm) causes a decrease of the RH from an initial level of 36% within minutes to a stable level below 20% whereas the temperature rises from room temperature of 19 °C to a stable level of about 30 °C. At this N_2_ flow rate, the PA signal shows an increase of about 1%, which has to be taken into account for a quantitative comparison of experimental data. The observed small fluctuations of T and RH at later times during long-term measurements influence the PA signal amplitude but can be compensated either mathematically or via a calibration of the sensor dependence on RH variations as discussed previously [35].

Further effects originating from the human body and the surrounding affect the PA signal recorded at the human forearm. This was demonstrated in a previous investigation when a periodical PA signal without laser irradiation was observed, which was caused by the blood pulsation of 55 heart beats per minute of the volunteer [29]. However, this PA signal is more than 100 times weaker than any laser-induced PA signal for identical measurement parameters. It is also significantly reduced at longer integration times of ≥1 s (as typically employed for glucose monitoring) and at higher modulation frequencies. Hence, the blood pulsation does not disturb the actual glucose PA measurements.

**Figure 5:**
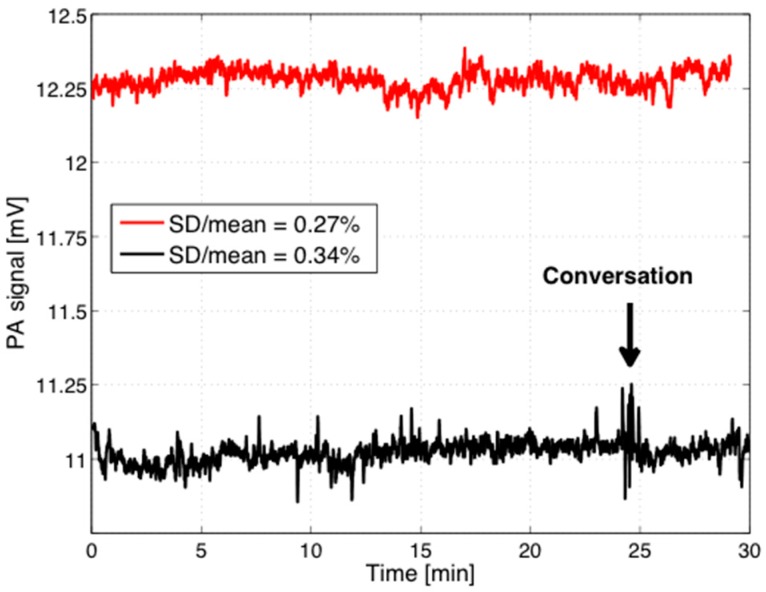
Stability of the PA signal recorded in vivo with the fiber-coupled PA sensor at the human forearm during two measurements events (red) and (black). Even a short conversation with the volunteer (indicated by the arrow) increases the PA signal fluctuations.

As a next step, the stability of the PA signal originating from human skin under laser irradiation was tested. Figure 5 shows the continuous recordings of the PA signal for two measurement events over 30 min. The ratio between the standard deviation (32 µV) and the mean value (12.25 mV) amounts to 0.26% for the upper trace. For the lower trace the ratio between the standard deviation (38 µV) and the mean value (11 mV) amounts to 0.34%. The arrow marks a noise-enhancement when the volunteer was speaking during the measurement indicating that silence and a minimum of movements should be obeyed during measurements to enable a low detection limit. However, if we assume a comparable PA signal dependence on the glucose concentration for in vivo measurements as obtained previously in vitro for aqueous solutions, a minimal detectable concentration of ≤50 mg/dL can be expected. This is lower than the corresponding limit of 100 mg/dL reported for skin samples [3], which is explained by the rather delicate measurements on the skin samples with a thickness of <100 µm. Furthermore, the pumping of the glucose solution and potential interfering substances explain the higher detection limit of those in vitro studies in camparison to the in vivo studies reported here. However, the minimal detectable concentration of 50 mg/dL is still a factor of about three higher than required for self-monitoring blood glucose devices according to the DIN EN ISO 15197 standard [36], but sufficient to detect in vivo glucose variations induced during a so-called oral glucose tolerance test (OGTT) as described below.

The naturally occurring blood glucose levels of a healthy human are limited to ~60–250 mg/dL. In order to induce high glucose levels in vivo, OGTTs were performed. For such tests, the healthy volunteers came to the laboratory in a fasting condition. Their preprandial glucose level was determined invasively with a commercial self-monitoring glucometer. All measured preprandial values were between 70 and 110 mg/dL, i.e., within the healthy range. Thereafter, the PA sensor was attached to their forearm as described above and the PA signal was continuously monitored during the entire OGTT. For these measurements, the EC-QCL was tuned to the distinct absorption peak of glucose at 1034 cm^−1^, the power was chosen close to the permissible irradiation level for skin and the modulation frequency kept at 137 Hz in order to attain deep skin layers but still fulfill the conditions of Equation (3). After a few minutes, when the PA signal was stable, a standardized glucose solution 300 mL of water containing 75 g glucose was drunk within ≤5 min at time “zero”. The drink caused a rapid increase of the glucose concentration in blood. Simultaneously to the continuous PA signal recording, the blood glucose concentration was monitored invasively by puncturing the finger tip of the volunteer every 10 to 20 min.

**Figure 6:**
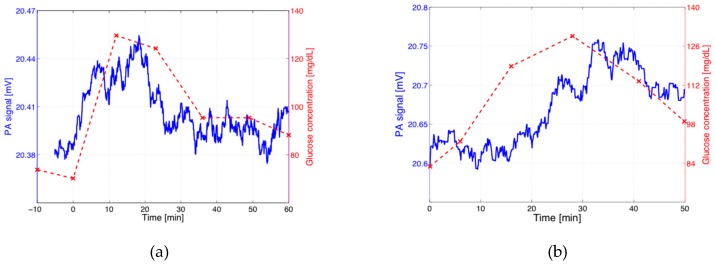
Continuously recorded “true” PA signals (blue) and invasively measured blood glucose (BG) data (dashed red) during an oral glucose tolerance test (OGTT) for two individual events (**a**) and (**b**). The “true” PA signals have been obtained after smoothing the original PA data (by applying a moving average of 10 s) and by rejecting fast PA signal changes caused by movements of the volunteer’s arm. Comparison of the non-invasive PA recording (blue) with the invasive measurement (dashed red) allows correlations to be found.

A typical result of a PA measurement is shown in Figure 6. Figure 6a depicts the conventional individual invasive measurements (in red) as well as the continuously recorded PA signal vs. time (in blue). In this case the RH correction was negligible because the RH fluctuations stayed below 0.3%. The original PA signal was smoothed by applying a moving average of 10 s. Furthermore, a filter was applied to suppress PA signal jumps caused by abrupt movements of the patient or variations of the PA cell-skin sealing. As Figure 6a implies, the “true” PA signal shows a delayed but distinct rise followed by a decay as expected from the invasive blood measurements. In this and other cases, a good correlation between the two measurements is observed. However, this qualitative agreement is not always observed as distinctly as in Figure 6a. An example with less pronounced correlation for an individual measurement at a fixed wavelength (1034 cm^−1^) is depicted in Figure 6b. The expected time delay between invasive and PA recording is absent (or even reversed), although one might argue that the peak blood glucose concentration for the invasive measurement might have been missed. Shorter time delays between invasive and non-invasive measurements could result depending on the skin thickness, skin composition and measurement site. These results demonstrate that the stability of the PA signal as well as the limited sensitivity (which is close to the expected glucose concentration changes during an OGTT) are the main problems encountered.

The stability issue is addressed with the experimental setup employing two QCLs at different wavelengths as discussed below.

### 4.2. Two QCLs at Fixed Wavelengths

#### 4.2.1. Glucose Measurement in Aqueous Solutions

**Figure 7:**
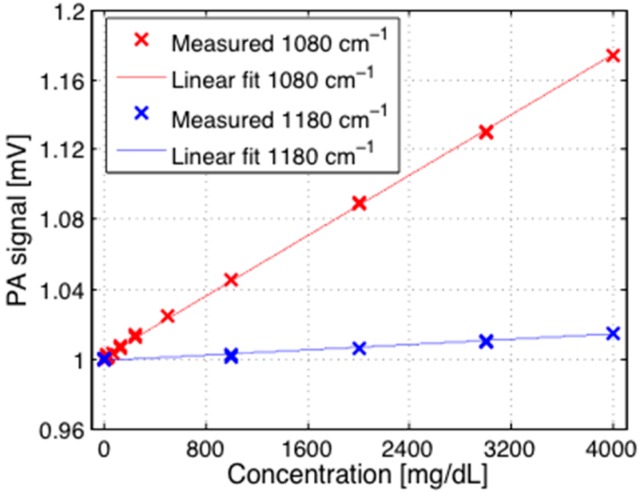
Dependence of the PA signal on the glucose concentration (in mg/dL) in aqueous solutions for two different wavelengths 1080 cm^−1^ (red) and 1180 cm^−1^ (blue) for a modulation frequency *f* of 137 Hz. The PA signals have been normalized to 1 for pure water.

The first measurement with the two QCLs concerns the dependence on the glucose concentration in aqueous solutions at two different wavelengths. These measurements were performed with the PA cell closed with a thin diamond membrane [23]. Figure 7 shows the results for 1080 cm^−1^ and 1180 cm^−1^. Obviously a linear dependence on the glucose concentration is obtained for the absorbing wavelength at 1080 cm^−1^ whereas an almost constant behavior results for the non-absorbing wavelength at 1180 cm^−1^. The different slopes observed for 1080 and 1180 cm^−1^ prove that the sensitivity to glucose is clearly wavelength-dependent and thus demonstrate the potential of the two-wavelength approach.

#### 4.2.2. Stability Measurements

**Figure 8:**
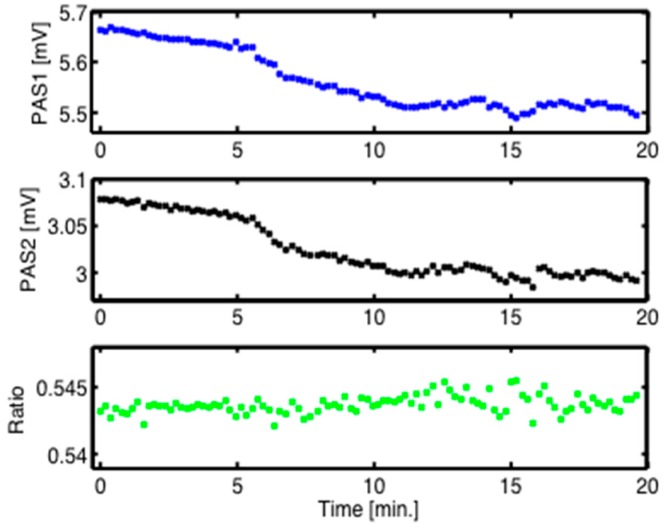
PA signals recorded at the fingertip of a healthy volunteer versus time with QCL 1 at 1180 cm^−1^ (PAS1: blue), with QCL 2 at 1080 cm^−1^ (PAS2: black), and ratio (green). Despite decreasing and fluctuating individual PA measurements, the ratio exhibits a rather constant signal.

Further measurements were devoted to the stability issue encountered in in vivo recordings as discussed above in Section 4.1. Experiments were performed with the N_2_-ventilated PA cell at the fingertip at the two selected wavelengths 1080 and 1180 cm^−1^, corresponding to a strong and a negligible glucose absorption, respectively. As Figure 8 implies, both measurements exhibit a decreasing signal with evolving time. However, the ratio is almost constant. This important result indicates that a gain in stability can indeed be achieved by using even only two selected wavelengths in a single measurement series, i.e., without any averaging. Based on the data of Figure 8, the signal variations calculated for the individual 20-min PAS measurements in comparison to their ratio are approximately eight times lower using the two-wavelength method compared to the single-wavelength scheme.

#### 4.2.3. Oral Glucose Tolerance Test (OGTT)

**Figure 9:**
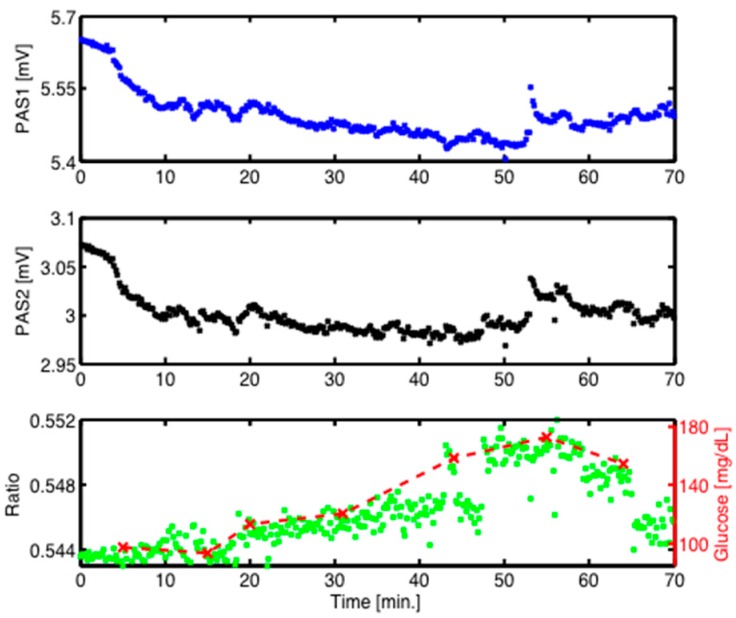
Continuous PA signals at 1180 cm^−1^ (PAS1: blue) and 1080 cm^−1^ (PAS2: black) recorded at the fingertip of a healthy volunteer during an oral glucose tolerance test (OGTT) as well as its ratio (green). A good correlation between the PA ratio signal (green) and simultaneous invasive blood glucose (BG) measurements (dashed red) is obtained.

Finally, a preliminary oral glucose tolerance test was performed as described above in Section 4.1, but now with two QCLs at the two fixed wavelengths 1080 and 1180 cm^−1^. In contrast to the OGTT measurements shown above, these measurements were recorded at the fingertip of the volunteer. The continuous PA signals vs time are plotted in Figure 9. The PA signal in Figure 9 is artificially shifted backwards by 15 min in order to match the maximum of the PA curve with the maximum of the invasively measured glucose content. This 15-min delay between the PA measurements and the actual blood glucose content is consistent with the time needed for the blood glucose to equilibrate with the interstitial fluid, from which the PA signal originates. Despite short-term signal fluctuations, the ratio of the individual measurements at the two wavelengths shows a clear rise and fall with evolving time. A maximum after approximately 55 min followed by a slow decrease is observed, as expected for an OGTT. The time delays between the invasive and continuous PA measurements are small, which can be explained by the measurement location at the fingertip. The rather large signal-to-noise ratios are due to the approximately 6 times lower laser powers employed in this case (as described above in Section 3.2), or 12 times lower if the “true” out-of-phase approach with simultaneous irradiation by two QCLs would be used. This result demonstrates that an individual time-resolved measurement during an OGTT can be reliably recorded by a dual-wavelength approach. It should be emphasized that neither data treatment nor data averaging were applied. Similar results have been obtained for several independent OGTT recordings.

**Figure 10:**
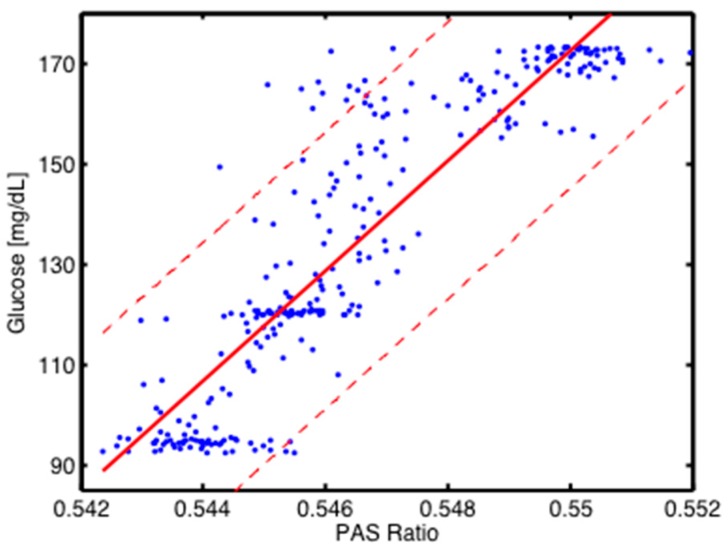
Correlation between invasive blood glucose data and non-invasive PA signals obtained with the 2-QCLs setup. The blue points are derived from the experimental data presented in Figure 9. The red solid line shows a linear fit (with r^2^ = 0.8), and the red dashed lines represent the confidence bounds at 90% confidence level.

The correlation between the invasively obtained blood glucose levels and the PA recordings is plotted in Figure 10. It has been derived from the measurements presented in Figure 9. The actual blood glucose contents are obtained using a cubic spline function on the invasive measurements (red X-symbols in Figure 9). The red solid line in Figure 10 shows a linear fit with r^2^ = 0.8. The confidence bounds with a 90% level of certainty are shown as red dashed lines in Figure 10. Based on the vertical distance between the confidence bounds, a blood glucose concentration uncertainty of ±30 mg/dL (at 90% confidence level) is derived.

## 5. Conclusions

We present mid-infrared QCL-based photoacoustic measurement schemes for glucose in human tissue. Both a fiber-based QCL-PA arrangement and a novel dual-QCL PA setup are discussed. Unlike conventional techniques the method is totally non-invasive. It does not record blood glucose directly but rather the glucose content in the interstitial fluid, which, however, is related to the blood glucose (BG) level with a time delay of ≤15 min at the used measurement sites. We tested the feasibility with an oral glucose tolerance test with healthy individuals. The measurements were performed with the PA cell in direct contact with the forearm for a continuous recording time of over 90 min. Simultaneously, the BG level was measured by puncturing the finger tip every 10 min and reading the BG level from a glucometer. The results indicate that the method with one QCL shows promising results but does not always yield a definite correlation with the BG data. First tests with two QCLs at fixed wavelengths selected for maximum and minimum glucose absorbance yield more reliable results despite six times lower laser power available. The dual wavelength scheme greatly improves the measurement stability, and a blood glucose concentration uncertainty of ±30 mg/dL is achieved at a confidence level of 90%. The detection sensitivity is still unsatisfactory for the physiological range of glucose concentration but it can be improved by employing higher laser power up to the recommended exposure limit for short-term irradiation. It should be emphasized that no advanced data treatment such as principal component analysis involving entire wavelength tuning ranges has been applied to actually demonstrate the feasibility under practical conditions, i.e., for continuous individual measurements. A further improvement is expected from studies involving more than two wavelengths characteristic for glucose absorption by involving several QCLs or even a QCL array with preselected fixed wavelengths. Finally, studies on diabetic patients need to be done to test the feasibility and to evaluate the potential of this diagnostic technique.

## Figures and Tables

**Table 1:** Material parameters of water and skin used for the calculation of the data displayed in Figure 2 [25,26,27].

Medium	Density *ρ* (kg/m^3^)	Thermal Conductivity *k* (W/m·K)	Specific Heat *C* (J/kg·K)	*α*_max_ between 1000 and 1100 cm^−1^ (cm^−1^)
Water	997	0.615	4181	651
Epidermis	1110–1190	0.293–0.393	3700	~ 0.6 · *α*_water_
Stratum corneum	1500	0.293–0.393	3700	~ 0.1 · *α*_water_
